# Metal dependent protein phosphatase PPM family in cardiac health and diseases

**DOI:** 10.1016/j.cellsig.2021.110061

**Published:** 2021-06-06

**Authors:** Chen Gao, Nancy Cao, Yibin Wang

**Affiliations:** a Division of Molecular Medicine, Department of Anesthesiology & Perioperative Medicine, David Geffen School of Medicine at University of California, Los Angeles, CA 90095, United States of America; b Molecular Biology Institute, David Geffen School of Medicine at University of California, Los Angeles, CA 90095, United States of America

**Keywords:** Protein phosphatas, Metal dependent, PPM, Signal transduction, Cardiac health, Cardiac disease

## Abstract

Protein phosphorylation and dephosphorylation is central to signal transduction in nearly every aspect of cellular function, including cardiovascular regulation and diseases. While protein kinases are often regarded as the molecular drivers in cellular signaling with high specificity and tight regulation, dephosphorylation mediated by protein phosphatases is also gaining increasing appreciation as an important part of the signal transduction network essential for the robustness, specificity and homeostasis of cell signaling. Metal dependent protein phosphatases (PPM, also known as protein phosphatases type 2C, PP2C) belong to a highly conserved family of protein phosphatases with unique biochemical and molecular features. Accumulating evidence also indicates important and specific functions of individual PPM isoform in signaling and cellular processes, including proliferation, senescence, apoptosis and metabolism. At the physiological level, abnormal PPM expression and activity have been implicated in major human diseases, including cancer, neurological and cardiovascular disorders. Finally, inhibitors for some of the PPM members have been developed as a potential therapeutic strategy for human diseases. In this review, we will focus on the background information about the biochemical and molecular features of major PPM family members, with emphasis on their demonstrated or potential roles in cardiac pathophysiology. The current challenge and potential directions for future investigations will also be highlighted.

## 1. Introduction

Protein phosphorylation/dephosphorylation is central to signal transduction and functional modulation, affecting virtually every process of cellular physiology. Protein kinases are responsible for adding the phosphate group on serine/threonine or tyrosine residues of their substrate proteins, leading to altered biochemical function, interaction, localization and stability, which are critical to cardiac pathophysiology [1–4]. Like all signaling processes, protein phosphorylation is dynamically and reversibly controlled not only by protein kinases, but also by their counteracting protein phosphatases, which are responsible for removing the phosphate group from targeted proteins. In the human genome, at least 518 possible protein kinases [5] and approximately 200 phosphatases [6,7] have been identified. For protein phosphatases, they are categorized into three major classes according to their substrate specificities, *i.e.*, tyrosine, serine/threonine (Ser/Thr) and dual-specific phosphatases [7,8]. Protein kinases are often viewed as the drivers of signal transduction attributed by their high specificity and robust regulation [9,10]. In contrast, protein phosphatases, in particular Ser/ Thr phosphatases, are often viewed as being constitutive and nonspecific, serving to balance and maintain signal homeostasis. Such bias may have contributed to the overwhelming imbalance in drug targets between kinases and phosphatases, with only a few exceptions.

Among the Ser/Thr phosphatases, three superfamilies have been identified based on their sequence and structure features [11], including phosphoprotein phosphatases (PPP), metal dependent protein phosphatases (PPM) and transcription factor II F (TFIIF)-interacting carboxyl terminal domain (CTD) phosphatases (FCP) [7,12]. FCP phosphatases are exclusively responsible for dephosphorylation of RNA polymerase II CTD [13]. On the other hand, the PPP phosphatases include PP1, PP2A and PP2B (calcineurin) subfamilies and have the broadest spectrum of substrates [14–16]. PPPs are oligomeric holoenzymes, composed of a handful of conserved catalytic subunits in complex with one or two regulatory subunits selected from a large pool of such proteins. It is believed that these regulatory subunits are essential for the diverse subcellular localization and substrate specificity carried out by the PPPs family [14–16]. In contrast, the PPM phosphatases are mostly monomeric enzymes [15,17]. Although relatively limited studies have been devoted to PPM phosphatases compared to the PPP family members, recent progress has identified many interesting roles for PPM isoforms in cellular stress response, growth signaling and metabolism [17,18]. The unique features of PPM phosphatases, the current knowledge of their biochemical properties, and their potential role in the context of cardiac health and diseases will be the focus of discussion here. Instead of striving for comprehensiveness, we aim to highlight the major gaps for future investigations and the potential therapeutic values for PPM family members.

## 2. PPM family: shared structural and biochemical features

PPM phosphatases are highly conserved in their sequences and structure with homologous genes identified from prokaryotes, animals and plants [17]. However, significant diversification occurred during evolution [17]. In mammals, a total of 18 functional and 2 enzymaticdead (pseudo-phosphatases) PPM isoforms are annotated [17]. Unlike PPPs, the PPM family members are largely monomeric enzymes with intrinsic sequence and structural features necessary for their targeted subcellular localization and substrate recognition. Most PPMs show Mg^2+^ or Mn^2+^ dependence for their catalytic activity, although Fe^2+^ has also been reported to activate while Cd^2+^ can inhibit some PPM activities [17].

**Fig. 1.**
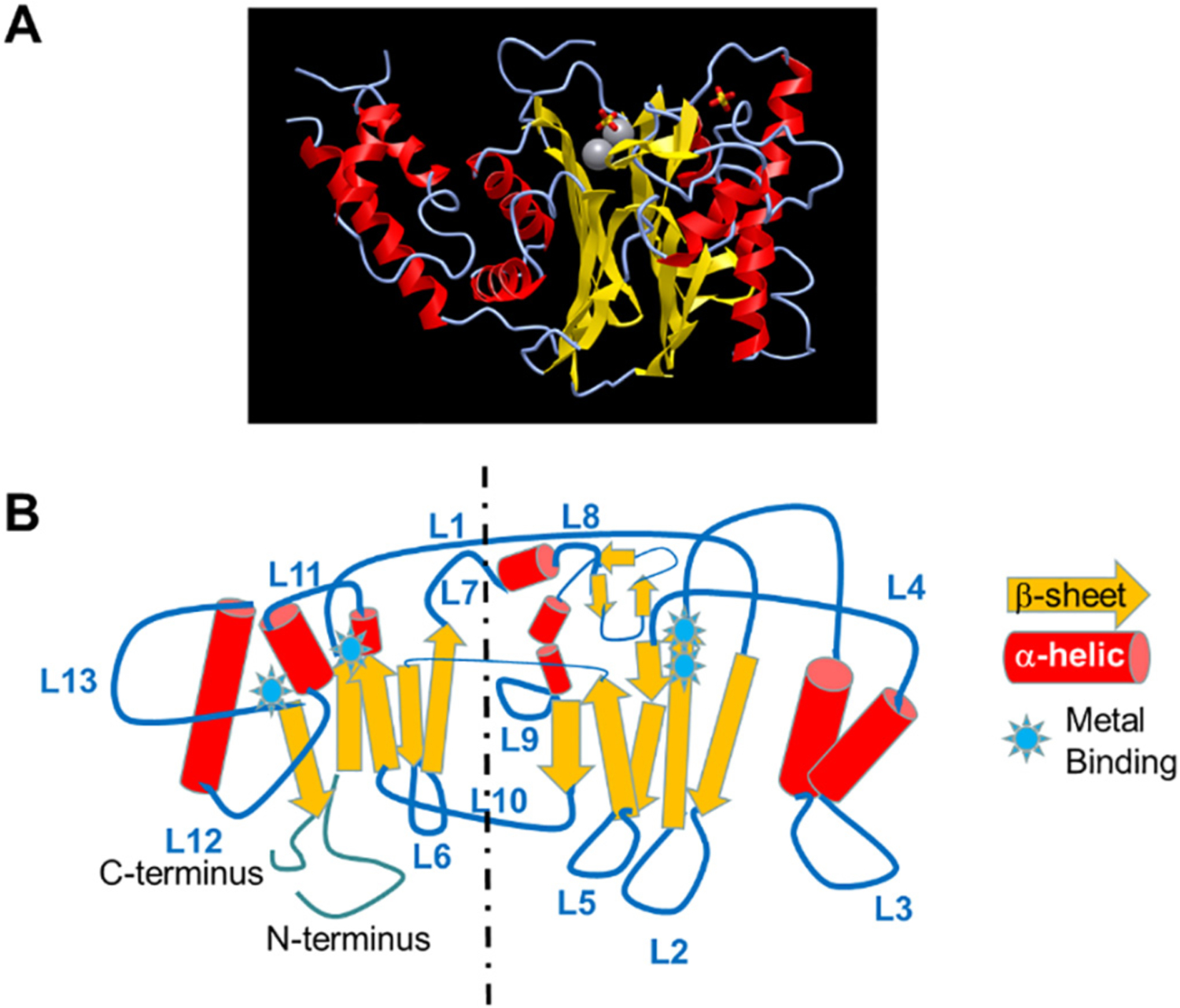
Structural features of PPM1 family. A. Crystal structure of PPM1A, extracted from https://structure.ncbi.nlm.nih.gov/icn3d/share.html?jT2XiUe6vY7NqXAp8. B. Illustration of PPM1 structure showing the distribution of β-sheet, α-helical, and 13 loops (L1–L13).

PPM family members all share a highly conserved catalytic core, even for the two pseudo-phosphatase members. Based on the known structure of human PPM1A [19], the catalytic core contains two β-sheets forming a sandwiched pocket, surrounded by four major α-helices. There are two metal chelating motifs embedded within the catalytic core for most PPM isoforms, which explains the metal-dependent activation for PPM family phosphatases (Fig. 1A). Beyond the conserved catalytic core, the 13 loops connecting the β-sheets and the α-helices in the catalytic core contain isoform specific features in sequence and lengths (Fig. 1B), which likely determine the unique biochemical features of each PPM isoform, including activation mechanism, substrate recognition and subcellular localization. A comprehensive review of the structure of PPM family members in the context of their biochemical properties has been published by Kamada et al. [17]. Although PPMs mostly function as monomeric enzymes, protein complex recruitment and enzymatic activities can also be regulated by post-translational modifications, including phosphorylation, oxidation and protein-protein interaction. While some mechanisms have been revealed for the selected isoforms, there is no overarching scheme of PPM activation that is applicable to all PPM isoforms. Therefore, it is possible that the structural diversity of PPM dictates the unique features of each isoform in function and regulation.

## 3. Functional spectrum of PPM family members

Despite their highly conserved molecular features, individual PPM isoforms participate in different cellular signaling processes with diverse features in substrate specificity, localization and regulatory interaction [17,18,20,21]. For simplicity, we will focus on selected members of the PPM family which have demonstrated potential roles in the cardiovascular system. In addition, the PHLPP subfamily will be discussed in more detail elsewhere in this special issue.

### 3.1. PPM1A and PPM1B in stress-signal transduction

PPM1A, also called PP2Cα, is one of the best characterized members of PPM family of phosphatase [22]. It is ubiquitously expressed [23] and is identified in complexes with stress-activated protein kinases (SAPKs) including p38 MAPK and c-Jun N-terminal kinase (JNK) [24], transforming growth factor-β (TGF-β) activated kinase (TAK) [25], NFκB [26] and Dvl/β-catenin/Axin complexes [27]. It can also dephosphorylate CDK9 T-loop [28], SMAD1/2/3 [29,30], ERK [31–33], antiviral response genes MAVS and SING [34,35], in addition to a number of other substrates. Accordingly, PPM1A can serve as a negative regulator for SAPK and NF-κB dependent stress responses, TGF-β activation and Wnt signaling [20]. In the PPM1A knockout mice, Smad1/2/3 phosphorylation is elevated; angiogenesis, inflammation and epithelial repair and regeneration during wound healing are impaired [36–38].

Although PPM1A and PPM1B share extensive similarities in sequence and structure, they also have distinct biological functions with both overlapping as well as unique substrates. In addition to the shared substrates of SAPKs, MEKK3 and IKKβ with PPM1A, PPM1B (or PP2Cβ) also dephosphorylates receptor-interaction protein kinase 3 (Rip3) [39] and proliferator-activated receptor-γ (PPARγ) [40]. Therefore, PPM1B contributes to the important processes of necroptosis, adipogenesis and inflammatory responses. PPM1B knockout mouse is embryonic lethal [41], highlighting its essential role in development and cellular homeostasis.

Despite the extensive literature reported on the substrate kinases in cardiac regulation, PPM1A and PPM1B are poorly studied in the context of cardiac physiology and pathology. Among these reports, PPM1A expression and activity are correlated with cardiac fibroblast activation induced by pathological stressors such as aldosterone or anti-fibrotic compounds including Simvastatin or Metformin [42,43], or cardiac lipid accumulation [44]. However, direct evidence supporting the role of PPM1A and PPM1B in cardiac pathophysiology have not been reported. Conceivably, however, PPM1A and PPM1B, by virtue of targeted regulation of stress activated MAP kinases and NFkB, can potentially affect cardiomyocyte hypertrophy and pathological remodeling. From its targeted regulation of Rip3 and PPARγ, we can also speculate that PPM1A/B may have a significant role in modulating myocyte viability, mitochondrial function and metabolic activities under stress conditions. Since these two PPMs are ubiquitously expressed, their cell type specific expression pattern should be carefully evaluated in intact heart and their potential roles need to be investigated with cell-type specific manipulation offered by current genetic tools.

### 3.2. Nuclear PPM1D and PPM1G in stress response and gene regulation

PPM1D (or wildtype p53-induced protein phosphatase 1, Wip1) is the most extensively studied member of the PPM family [45]. PPM1D is specifically located in the nucleus potentially through two conserved nuclear localization motifs [46]. It has strong substrate preference towards the pT-X-pY motif, a signature feature found in the activation/ regulatory lip across all three branches of the MAP kinases [47]. Phosphorylation and dephosphorylation of the pT-X-pY motif serves as an essential switch for MAP kinase activation/inactivation by upstream MAP kinase kinases (MKKs) and protein phosphatases [48]. While PPM1D is not the only protein phosphatase to have such activity, it might have a significant role in MAP kinase mediated transcriptional regulation in the nucleus. One prominent substrate of PPM1D is ataxia telangiectasia mutated (ATM) kinase, which is a central player in DNA damage response and p53 activation [49]. Tempering ATM activity and p53 activation is a common molecular scheme shared by many cancer cells. PPM1D amplification or gain-of-function mutations have been reported in different types of cancer [50–52]. PPM1D knockout mice develop immune disorders with major impacts on T- and B- cell development, neutrophil differentiation, and macrophage activation in association with disrupted p53, mTOR or p38 signaling [53], but are resistant to cancer [54]. In addition to ATM mediated cell-cycle arrest, PPM1D also regulates proliferation through other check-point proteins, including CHK2, HIPK2, H2AX and H2AZ. Finally, PPM1D also modulates PPARγ phosphorylation and activity, as well as autophagy regulator Unc-51-like kinase (ULK) in adipose tissue, and thus participate in adipogenesis, lipid homeostasis and atherosclerosis formation [55,56]. In short, PPM1D is a nuclear protein phosphatase engaged in stress-signaling, DNA damage response, and transcription regulation, with important roles in cell-differentiation and proliferation.

Much of the current literature on PPM1D focuses on its role in p53 regulation and DNA damage response in cancer. However, p53 mediated transcription reprogramming and DNA damage response are common molecular signatures in heart failure caused by ROS injury, ischemia/reperfusion and anti-cancer therapies [57,58],. Abnormal activation of p53 mediated signaling and DNA damage response have also been found in arrhythmogenic cardiomyopathy (ACM) caused by LaminA/C and Tmem43 deficiency [59–61]. Therefore, it is surprising that very limited study has been devoted to PPM1D’s function in the heart. Consistent with its role as an inhibitor to stress/injury induced signaling, Liu et al., reported that PPM1D knockout mice showed exacerbated injury following myocardial infarction [62]. Finally, PPM1D mutation is also a common genetic lesion associated with clonal hematopoiesis during aging and cancer [63,64]. There is growing recognition that clonal hematopoiesis represents an important mechanism of abnormal inflammation in heart failure, particularly associated with aging [65]. Therefore, a direct role for PPM1D in cardiac inflammation may be worth investigating. Overall, PPM1D, by regulating genome integrity, could play a significant role in ischemia-reperfusion injury, postinfarction remodeling, arrhythmogenic cardiomyopathy, cardiac complication associated with cancer therapy, and aging related chronic heart failure.

PPM1G is another nuclear enriched PPM family member which interacts with and modulates Cajal bodies and survival motor neuron complex (SMN) to regulate RNA splicing machinery as part of the spliceosome complex [66]. The molecular basis of PPM1G nuclear targeting is not clear. Loss of PPM1G in mice led to early embryonic lethality with major neural developmental defects [67]. However, its role in cardiac physiology and disease has not been reported. Considering the importance of RNA processing in cardiomyopathy and heart failure, it would be highly informative to explore its function in the context of cardiac development and disease progression under pathological stresses.

### 3.3. Mitochondrial PDPs and PPM1K in metabolic regulation

PDP-1, PDP2 (Pyruvate dehydrogenase phosphatase 1 and 2) are located in the mitochondrial matrix through conserved mitochondrial targeting pre-sequence at their N-terminal which is cleaved from the mature forms [68,69] a conserved feature among mitochondrial matrix proteins [70]. Unlike other PPM family members which function as monomeric enzymes, the PDPs are the catalytic subunits which form heterodimeric enzymes with a regulatory subunit (PDPR). As indicated by their names, PDPs are highly specific pyruvate dehydrogenase (PDH) phosphatases, helped by the specific tethering between PDPs and the lipoyl motif of the PDH E2 subunit through the regulatory subunit PDPR [68]. PDH is the rate limiting enzyme in pyruvate catabolic pathway leading to Acetyl-CoA production and is an essential link between glycolysis and ATP production and fatty-acid biosynthesis. PDH activity is potently regulated by phosphorylation and dephosphorylation of its E1α subunit. PDH kinases (PDKs) are responsible for PDH-E1α phosphorylation and inactivation. On the other hand, PDPs activate PDH activity by removing E1α phosphorylation. PDKs have been extensively studied, and the altered expression and activities of PDKs have been implicated in metabolic flux regulation, Warburg effect and mitochondrial respiration [68,69]. Importantly, different from other PPM isoforms, PDPs show marked Ca^2+^ dependent induction of their activities in addition to Mg^2+^/Mn^2+^ dependence [71,72]. This feature could be an important molecular basis linking adrenergic and metabolic signaling (such as catecholamine and insulin stimulation) and mitochondrial activities, since PDP activity promotes pyruvate utilization to fuel TCA cycle and ATP production [69,72,73]. Defects or abnormal activation/ inactivation of PDKs have been extensively studied in a broad list of human diseases, including diabetes, cancer and neurological defects. In stark contrast, PDPs in heart have received limited attention so far. A few reports show the expression and activities of PDPs are correlated with hypertrophy and aging in the heart and can impact on cardiomyocyte differentiation [74–76]. But the direct and specific impact of abnormal activity or expression of PDPs in cardiac development, pathogenesis of diabetic cardiomyopathy, ischemic injury and pathological remodeling remain to be explored.

PPM1K is another mitochondrial targeted PPM member owing to the presence of a mitochondrial targeting pre-sequence at its N-terminus, and its substrate has been demonstrated to be the branched-chain α-ketoacid (BCKA) dehydrogenase complex (BCKD) [77,78]. Interestingly, BCKD is a genetic duplicate of PDH and catalyzes the rate limiting step of branched-chain amino acid (BCAA) catabolism [79]. Parallel to the regulatory scheme of PDH, BCKD activity is also potently regulated by phosphorylation and dephosphorylation of its E1α subunit by BCKD kinase (BCKDK) and PPM1K. BCKD deficiency is causal to Maple-Syrup Urine Disease (MSUD), characterized by abnormal accumulation of BCAA/BCKA and lethal neurological complications. More recently, BCAA defects have also been implicated in autism, cancer, obesity, diabetes and heart failure by modulating nutrient sensing pathways such as mTOR and insulin, as well as ROS and glutamine homeostasis [80]. Loss of PPM1K in human is associated with a mild form of MSUD [81] while reduced expression of PPM1K is associated with heart failure, obesity and insulin resistance in both human samples and animal models [82,83]. In animal models, loss of PPM1K expression promoted while PPM1K over-expression attenuated heart failure induced by pathological stressors (including pressure-overload and myocardial infarction) and metabolic challenge [82,84]. Although BCAA catabolic defects appears to be an important metabolic signature in the diseased heart [85], the underlying mechanism remains elusive [85–87]. A recent report also shows PPM1K and BCKDK can directly regulate cytosolic ATP-citrate lyase, which potentially integrates BCAA catabolism and lipid metabolism [80,88]. Therefore, the non-canonical activities of PPM1K should be carefully considered as well. In summary, PDPs and PPM1K are both mitochondrial isoforms of the PPM family regulating glucose and BCAA metabolism by targeting their corresponding key steps of catabolism. There is emerging evidence to implicate their contribution to cardiac development and metabolic health. However, their full involvement in cardiac metabolic regulation under normal development and disease progression have just begun to be recognized.

### 3.4. Membrane associated PPM1L, PHLPP1 and PHLPP2 in local cell signaling

PPM1L is an ER targeted PPM isoform due to its transmembrane motif in its N-terminal region [21]. In addition to TAK1 as a shared substrate with PPM1A and PPM1B, PPM1L specifically dephosphorylates IRE1 [89], a branch of the ER stress response pathway, and functions to attenuate unfolded protein response mediated by IRE1 activation. Uncontrolled IRE1 activation has been linked to cell death and PPM1L knockout female mice showed defective lactating capacity in their mammary gland and lactation-induced cell death in mammary epithelium [89]. PPM1L is identified as a candidate gene affecting metabolic regulation through systems genetic approach [90] and is shown to be necessary for normal adipocyte maturation related to IRE1 mediated signaling [91]. In cardiomyocytes, PPM1L also targets the CaMKII phosphorylation site of phospholamban (PLB) [92]. Upon overexpression in mouse hearts, PPM1L exacerbated ischemia-induced cardiac dysfunction, presumably by tempering either cytoprotective ER stress response or PLB mediated calcium homeostasis. It is important to note that PPM1L has remarkable substrate specificity. While it potently suppresses IRE1 auto-phosphorylation, it has no impact on PERK phosphorylation, which is also an ER membrane associated signaling branch in unfolded protein response [91]. In addition, PPM1L specifically dephosphorylates CaMKII dependent Thr-17 phosphorylation on PLB but shows no effect on the neighboring PKA dependent Ser-16 phosphorylation [92]. This level of specificity defies the traditional view of Ser/Thr phosphatases downstream target specificities. However, the molecular basis of such specificity is unknown, and it is likely that PPM1L may have other downstream targets in a cell type specific manner and serves as a potent regulator for ER-membrane associated local signaling. Giving the important role of PPM1L in ER stress and SR calcium regulation, it would be expected that PPM1L can potentially play a role in regulating cardiac stress response and contractile regulation, particularly under pathological conditions.

PHLPP1 and PHLPP2 are two PH-domain containing PPM isoforms with targeted localization to cytoplasmic membrane [17]. They have been extensively studied in cardiac hypertrophy and signaling by Purcell lab, and will be discussed in detail elsewhere in this issue [93–96].

### 3.5. Other PPM isoforms

PPM1E and PPM1F, also known as POPX1 and POPX2, are two highly homologous PPM isoforms [17]. PPM1F is ubiquitously expressed, while PPM1E is more restricted to brain and testicular tissue. PPM1E and PPM1F bind to and dephosphorylate p21-activated protein kinase (PAK) and calcium calmodulin dependent kinase (CaMK) [97]. However, there are limited studies on their function *in vivo* based on genetic manipulations. PPM1H is a cytosolic PPM isoform with known phosphatase activities towards SMADs 1/5/8, Rab and p27 [98–102]. Beyond some indication of its role in BMP signaling and tumor resistance, its function in cardiovascular system is unknown. Finally, ILKAP (Integrin-linked Kinase Associated Protein) was originally identified as a binding partner of ILK and negatively regulates ILK signaling to downstream GSK-3β activation [103–105]. More recently, it has been reported that ILKAP targets HIF-1α and has an essential role in hypoxia induced apoptosis [106]. Although both integrin signaling and hypoxia are highly relevant in cardiac pathophysiology, the functional significance of ILKAP-mediated regulation in the heart has not been reported. It can be speculated that these PPM family members can potentially regulate TGF-β signaling and mechanical sensing, thus contributing to cardiac remodeling in response to pressure or volume overload.

## 4. PPM: potential new therapeutic targets for heart diseases

**Fig. 2.**
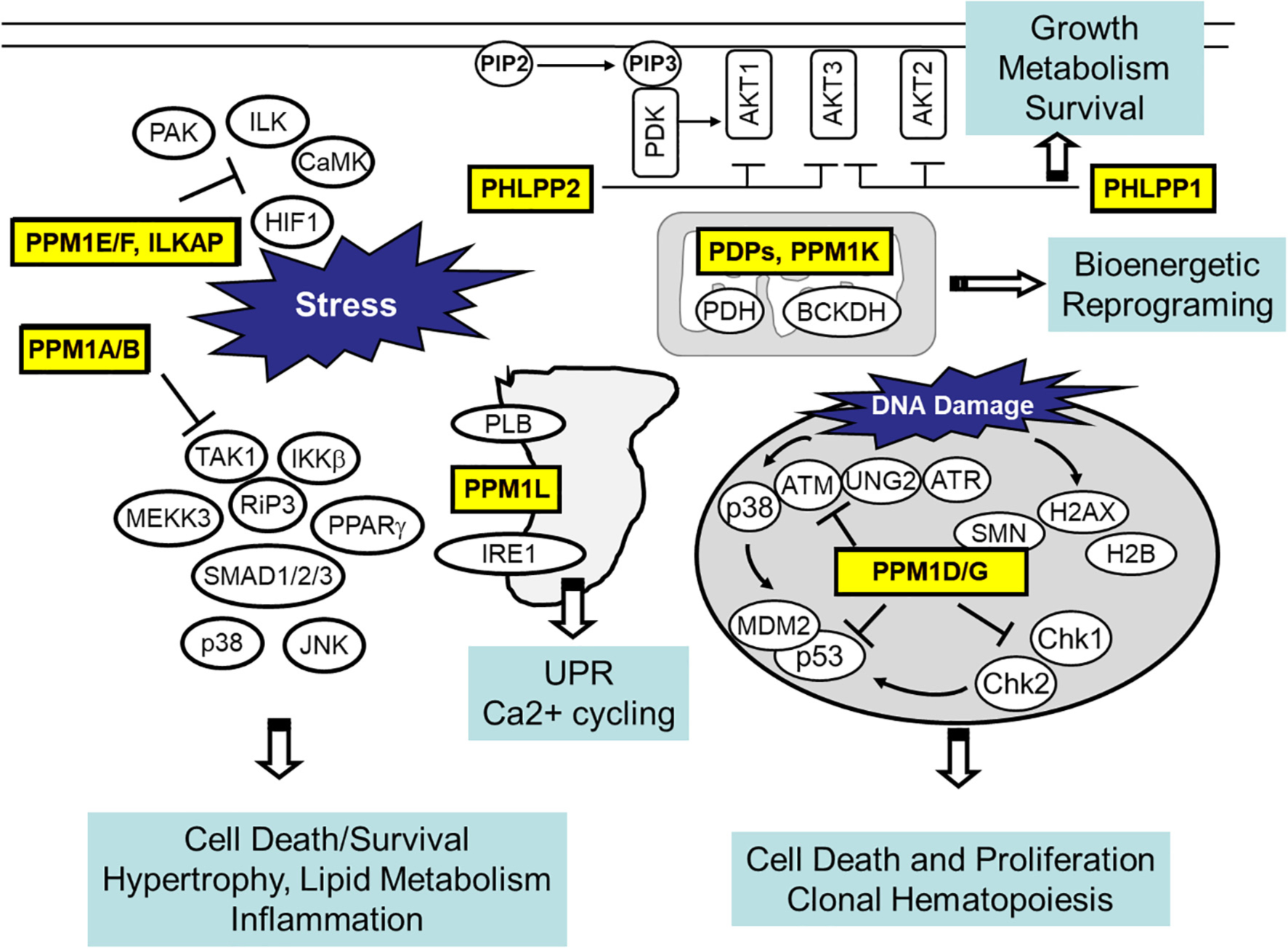
Intracellular localization, targets and potential downstream cellular effects for major PPM isoforms.

**Fig. 3.**
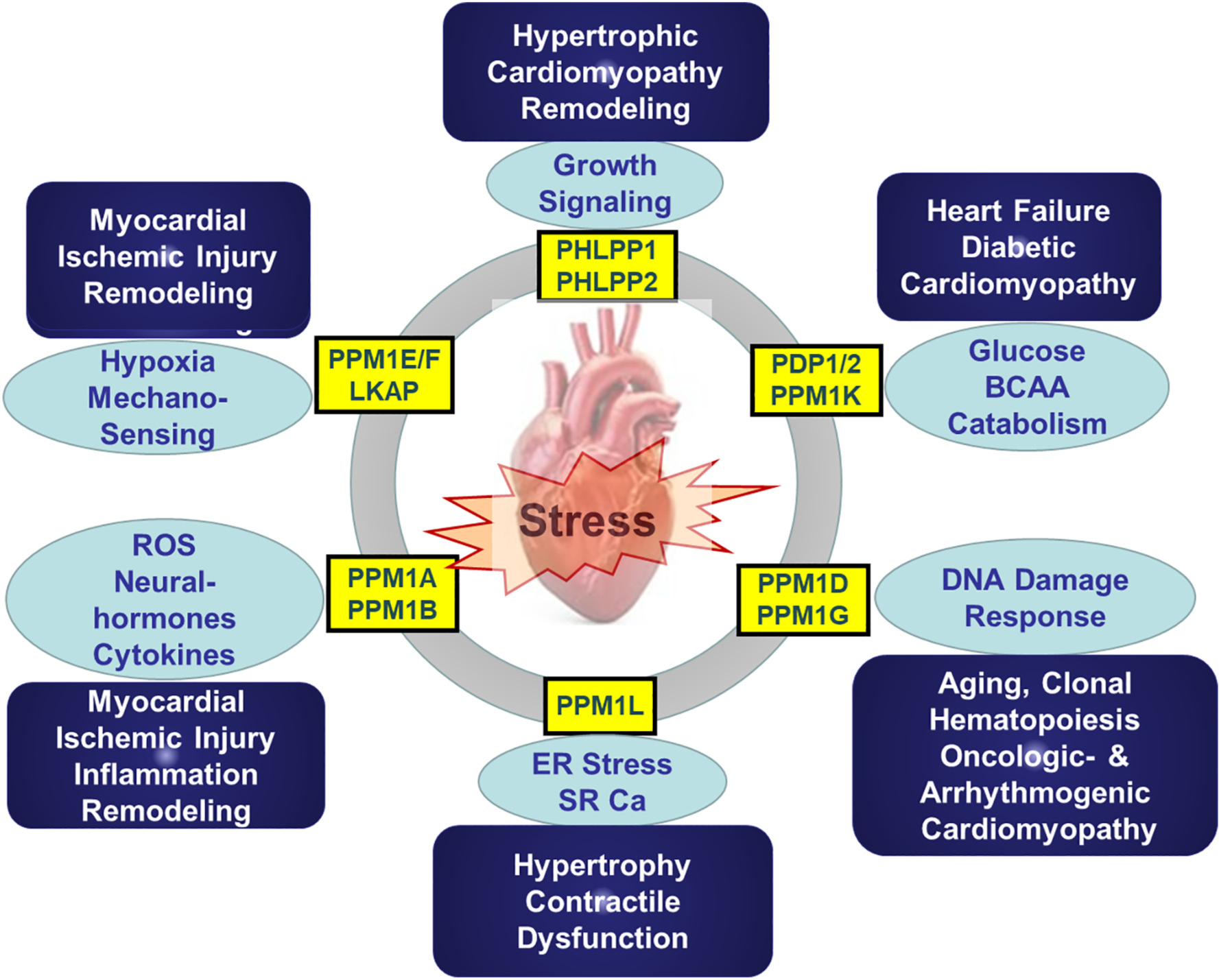
Potenital targeted signaling pathways and pathological implications for individual PPM family members. ROS, reactive oxygen species; ER, endoplasmic reticulum; SR-Ca, sarcolama calcium.

As discussed in detail above, the PPM family of Ser/Thr protein phosphatases consists of functionally diverse members. Although they share a highly conserved catalytic core, each member demonstrates remarkable specificity in either substrate recognition or intracellular localization (Fig. 2). While much of the insights about PPM function have been established in cancer, neural and other cell systems, more evidence is emerging for their relevance in cardiac physiology and pathology. Considering the known pathways involved in PPM mediated regulation, including stress-signaling, cell death regulation, DNA-damage response, fuel-specific metabolism and ligand induced extracellular signaling, it is highly likely that they may play important roles in cardiac response to pathological stress and injuries. However, except for a handful of examples, majority of the PPM family members have not yet been well studied in the heart, arguing for a great need for future investigations. At the fundamental level, PPM mediated substrate targeting has remarkable specificity, but the molecular basis remains to be decoded which will allow substrate specific interference. Tissue-specific role in heart should be better investigated with the emerging single cell-based transcriptome or proteome analysis platforms. Inactivation or activation (loss- or gain-of function) studies need to be implemented in specific cardiac constituents (myocytes, endothelium, fibroblasts, macrophage et al.) using targeted genetic manipulations. In particular, stress-induced cardiac hypertrophy, dysfunction, fibrotic scar formation and metabolic disorder should be investigated more specifically as potential outcomes of PPM mediated regulation in the heart (Fig. 3).

One challenge to investigating PPM function and translating the outcome to cardiac physiology and diseases is the lack of PPM specific pharmacological reagents. Unlike protein kinases, there is a paucity of available inhibitors or agonists targeting protein phosphatases, especially for PPM family members. In the past decade, several isoform specific inhibitors have been reported, mostly targeting PPM1A, PPM1D, PHLPP1/2 and PPM1E/F isoforms and have been tested as potential cancer therapy [17]. However, substrate selectivity for many of these compounds have not been fully demonstrated, and their IC50s are mostly in micromolar range with only a few PPM1D inhibitors in nanomolar range. As more structural information of PPM isoforms have become available, efforts to develop PPM pharmacological reagents based on in-silico modeling becomes feasible, and new classes of PPM-manipulating small molecules may become available in the future.

Earlier studies demonstrate that PPM1K competes with BCKDK to interact with their shared binding motif on BCKDH E2 subunit and confer dephosphorylation or phosphorylation of the E1α subunit [77,78]. But specifically inhibiting BCKDK using an allosteric inhibitor to block BCKDK interaction with E2 allows PPM1K mediated activation of BCKDH activities [107]. Indeed, PPM1K knockout exacerbates while inhibition of BCKDK interaction with BCKDH confers potent amelioration to pressure-overload induced heart failure [82,85], highlighting the potential of PPM targeted manipulation as a viable therapeutic strategy for heart failure. The functional landscape of PPM in cardiovascular physiology and diseases remains a vastly under-explored frontier in signal transduction. With continuing success in genetic and pharmacological manipulation, we can expect more progress in this exciting area of research in the coming years.
